# Supplementary material for: Investigating the Molecular Mechanisms Underlying Early Response to Inflammation and Helicobacter pylori Infection in Human Gastric Epithelial Cells
Source: Int J Mol Sci. 2023 Oct 13;24(20):15147. doi: 10.3390/ijms242015147 (PMC10607124; doi:10.3390/ijms242015147)
Supplement: Supplementary file 1 [file ijms-24-15147-s001.zip › ijms-2624503-supplementary.pdf]

Article

"Investigating the molecular mechanisms underlying early response to inflammation and *Helicobacter pylori* infection in human gastric epithelial cells"

Martinelli et al., 2023

Supplementary data

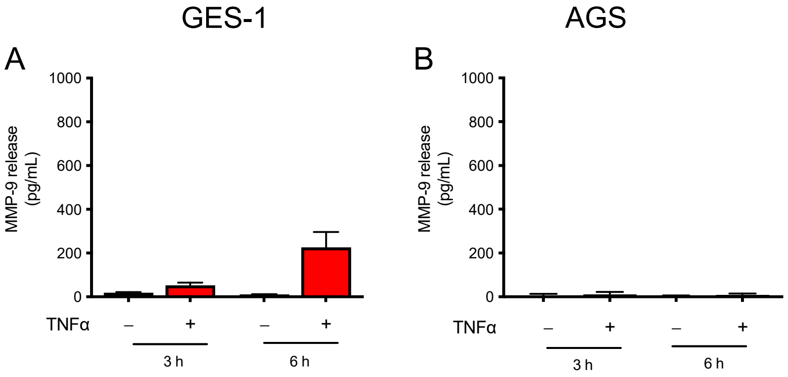

Figure S1. MMP-9 release to TNF in GES-1 and AGS cells.

GES-1 (red bars) and AGS (black bars) cells were treated for 3 and 6 h with TNF (10 ng/mL). MMP-9 secretion (A, and B) was measured by ELISA. Results are expressed as mean ± SEM of at least 3 experiments performed in duplicates.

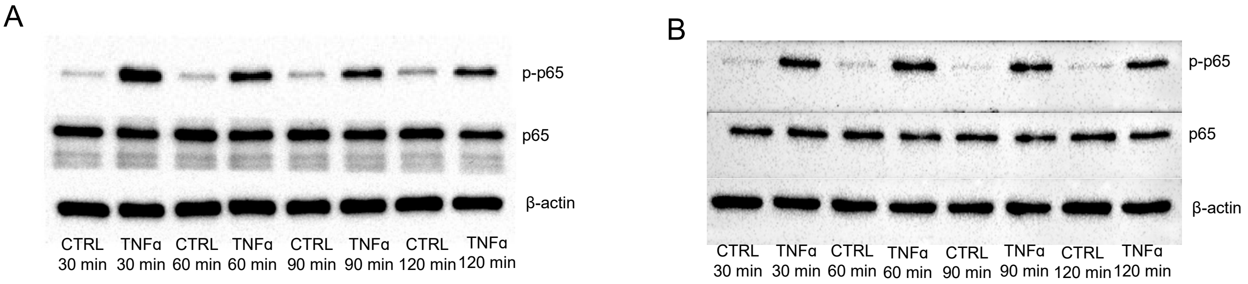

Figure S2. NF-κB signaling in TNF treated GES-1 and AGS cells.

Representative blots for the phosphorylation state of p65 (p-p65) of GES-1 (A) and AGS (B) cells treated with TNF (10 ng/mL) for the indicated time points.

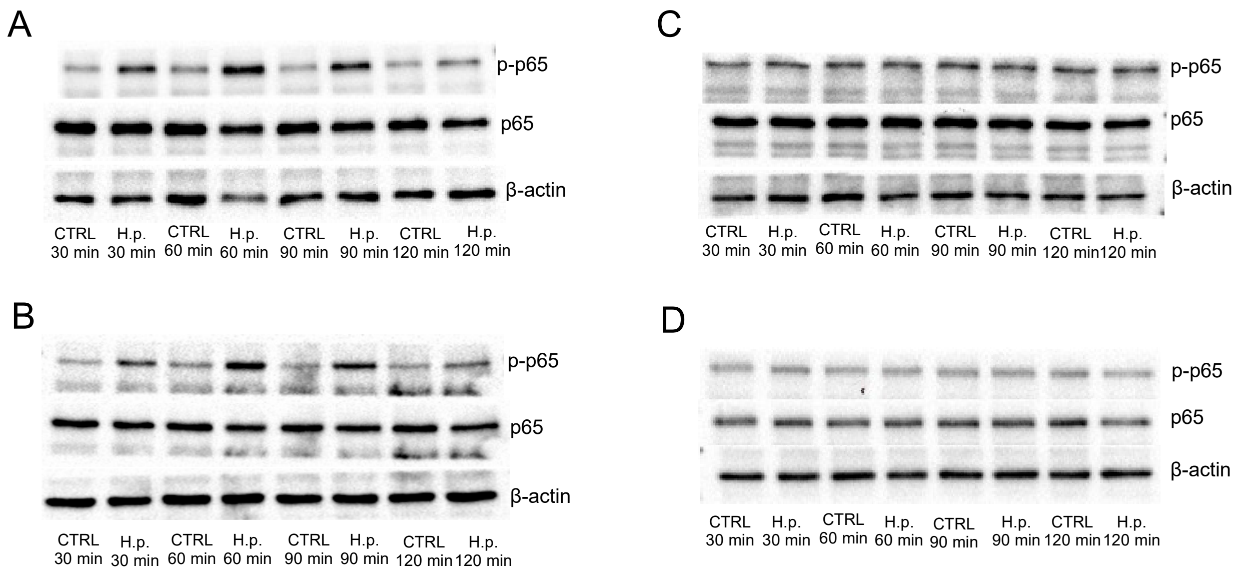

Figure S3. NF-κB signaling in GES-1 and AGS cells infected with *H. pylori* strains.

Representative blots for the phosphorylation state of p65 (p-p65) of GES-1 (A,C) and AGS (B,D) cells treated with *H. pylori* strains (bacterium-to-cell ratio, 50:1) for the indicated time points. A) and B) Cells were infected with the CagA-positive (ATCC) and C) and D) with CagA-negative clinical isolate #6 (#6) strain.

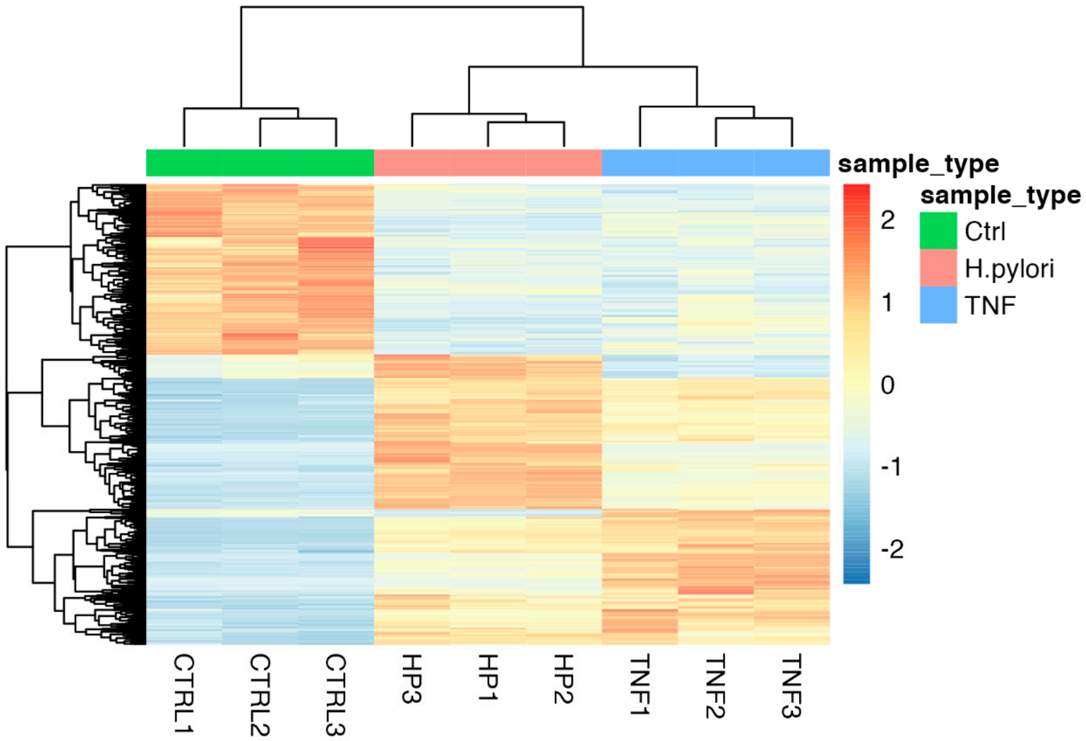

Figure S4. Transcriptomic analysis of *H. pylori*-infected and TNF -treated GES-1 cells vs untreated cells

Heatmap representing the distribution of differentially expressed genes (DEGs) in infected (*H. pylori*) or TNF-treated (TNF) cells in respect to untreated cells. GES-1 cells were infected with the CagA-positive (ATCC) *H. pylori* strain (bacterium-to-cell ratio, 50:1) or treated with TNF (10 ng/mL) for 6 h. RNA-seq was performed as described in Materials and methods.

Supplementary table S1. Virulence and antibiotic resistance of the two strain of *H. pylori* included in this study (ATCC strain , and clinical isolate #6)

H. pylori strain	Antibiotic resistance	Antibiotic sensitivity	CagA	Allelic combination of VacA
ATCC strain	Claritromycin/metronidazole	Levofloxacin/amoxicillin	+	S1/M1
#6	Claritromycin/metronidazole	Levofloxacina/amoxicillina	-	S2/M2

Supplementary table S2. Differentially expressed genes following 6 h *H. pylori* infection (ATCC strain) (Human Inflammatory Cytokines & Receptors RT² Profiler PCR Array)

	GES-1		AGS		
Gene symbol	Nominal p value	Fold Change	Nominal p value	Fold Change	NF-κB Target*
BMP2	>0.05	NA	$4.74x10^{-2}$	3.256	Yes
CCL20	>0.05	NA	$5.13x10^{-3}$	201.784	Yes
CCL3	>0.05	NA	$2.38x10^{-2}$	3.7754	Yes
CXCL2	>0.05	NA	$1.79x10^{-2}$	3.706	Yes
CXCL8	>0.05	NA	$6.26x10^{-4}$	28.907	Yes
IL6	>0.05	NA	$1.88x10^{-2}$	5.442	Yes
SPP1	>0.05	NA	$3.09x10^{-2}$	0.569	yes
TNFSF10	>0.05	NA	$2.21x10^{-2}$	0.095	Yes

*Data extrapolated according to the Thomas Gilmore's lab list [17] and the NF-κB target gene sets [18]. NA, not applicable, indicates that expression was not significantly changed by the treatment

Supplementary table S3. Differentially expressed genes following 6 h *H. pylori* infection (#6 strain) (Human Inflammatory Cytokines & Receptors RT² Profiler PCR Array)

	GES-1		AGS		
Gene symbol	Nominal p value	Fold Change	Nominal p value	Fold Change	NF-κB Target*
C5	$7.19x10^{-3}$	0.207	>0.05	NA	No
CCL26	$1.68x10^{-2}$	0.285	>0.05	NA	No
CCL7	$7.98x10^{-3}$	2.770	>0.05	NA	Yes
CSF3	>0.05	NA	$4.74x10^{-2}$	4.307	Yes
IL6	>0.05	NA	$2.56x10^{-2}$	3.627	Yes
MMP9	$4.91x10^{-3}$	2.147	>0.05	NA	Yes

*Data extrapolated according to the Thomas Gilmore's lab list [17] and the NF-κB target gene sets [18]. NA, not applicable, indicates that expression was not significantly changed by the treatment

Supplementary table S4. Top enriched pathways with upregulated or downregulated genes in TNF-treated in comparison to untreated cells

Term	Genes
**Enriched pathways with upregulated genes**	
cytokine-mediated signaling pathway (GO:0019221)	*CSF1; IFIT5; TNF; IFIT3; ICAM1; IL27RA; MT2A; PIM1; TRIM25; MAP3K8; JUNB; TNFRSF4; TRIM21; IFNAR2; IL15RA; IL4R; IL1R1; PRKCD; PRLR; IL6ST; SQSTM1; TP53; CARD14; IFNAR1; EBI3; TNFRSF11B; HIF1A; SOCS3; UGCG; SOCS1; IRAK2; MCL1; STAT5A; TNFSF18; VCAM1; TNFSF14; TNFSF15; TNFRSF9; LIF; NFKB1; ISG20; NFKBIA; CXCL10; CXCL11; IL6; BCL6; TNFSF4; IL7R; CXCL6; CDKN1A; CIITA; CD40; ITGAM; CXCL8; PIK3CD; CXCL1; CXCL3; CXCL2; CX3CL1; TRIM8; JAK1; SERPINB2; IFNGR1; RIPK2; IL15; IFNGR2; MMP3; TRAF2; TRAF1; TNFRSF1B; OSMR; MMP9; IL1A; CEACAM1; TRAF3; IL1B; IRF1; OAS3; IL3RA; IRF2; IRF7; LTA; LCP1; LTB; BIRC2; BIRC3; FOXC1; CEBPD; NOD2; PTGS2; IL2RG; RELA; PSMB10; MUC1; CCL5; CCL2; STAT6; STX4; GBP2; SH2B3; GBP1; IL32; CCL20; STAT1; CD70; IL34; STAT3; SOD2; PML; PSMB9; TNIP2; SAA1; TRIM31*
cellular response to cytokine stimulus (GO:0071345)	*CDKN1A; CD40; ITGAM; CXCL8; CSF1; PIK3CD; MT1X; CXCL1; TNF; CXCL2; YBX3; CX3CL1; ICAM1; IL27RA; ZFP36; MT2A; PIM1; SOX9; JUNB; JAK1; NKX3-1; IFNAR2; GBP5; IL4R; IFNGR1; IL1R1; IFNGR2; MMP3; TNFRSF1B; OSMR; PRLR; MMP9; IL1A; IL1B; IRF1; IL3RA; CD47; IL6ST; TP53; TNFRSF21; IFNAR1; FOXC1; SELPLG; CEBPD; EBI3; PTGS2; IL2RG; HIF1A; TANK; RELA; SOCS3; SBNO2; MUC1; SOCS1; IFI16; IRAK2; CCL5; CCL2; STAT6; STX4; GBP2; SH2B3; GBP1; GBP4; MCL1; GBP3; STAT5A; IL32; XBP1; SMAD3; VCAM1; CCL20; STAT1; IL34; STAT3; LIF; NFKB1; CXCL10; IL6; BCL6; SAA1; PTPN2*
regulation of inflammatory response (GO:0050727)	*PTGER4; SERPINE1; TNFAIP3; AOAH; NOD2; MEFV; PTGS2; ETS1; TNF; CX3CL1; RELA; CDH5; SBNO2; CCL5; CASP4; IL1R1; IL15; SPHK1; PRKCD; MMP3; NMI; TNFRSF1B; MMP9; NFKBIA; CYLD; MFHAS1; GPRC5B; IL6; BCL6; TNIP1; TNFSF4; IL1B; NINJ1; SAA1; TLR7; CD47; BIRC2; PTPN2; PTGES; BIRC3*
inflammatory response (GO:0006954)	*PTGER4; CXCL6; CD40; CIITA; CEBPB; CXCL8; PIK3CD; ADM; CXCL1; MEFV; CXCL3; TNF; CXCL2; HDAC9; CX3CL1; RELA; RELB; HRH1; CCL5; BDKRB2; OLR1; CCL2; RAC1; TNFRSF4; APOL3; AFAP1L2; RIPK2; CCL20; NFAM1; STAT3; PLA2G4C; NFKB1; IL1A; CXCL10; MFHAS1; CXCL11; IL6; ELF3; KRT16; TNFSF4; IL1B; PTX3*
cellular response to lipopolysaccharide (GO:0071222)	*CD274; CXCL6; CXCL8; SERPINE1; TNFAIP3; CXCL1; NOD2; CXCL3; TNF; CXCL2; RELA; ZFP36; SBNO2; CCL5; CCL2; LYN; XBP1; PDCD1LG2; TICAM1; TNFRSF1B; NFKB1; CXCL10; IL1A; CXCL11; IL6; TNIP2; TNFSF4; IL1B; TNIP3; NFKBIB*
regulation of cell population proliferation (GO:0042127)	*BTG4; BTG3; CDKN1A; ATP8A2; CXCL8; CSF1; PDCD5; SIX1; CXCL1; LAMC2; AREG; ETS1; IFIT3; CX3CL1; RERG; ESM1; SIX4; FTH1; NAMPT; CYP1B1; SOX9; IER5; JUNB; BOK; NKX3-1; SERPINB3; KLF10; EDN1; IL15; RHOG; NRG1; OSMR; SSTR2; PRLR; RUNX1; IL1A; IL1B; IRF1; TRNP1; CD47; IL6ST; TP53; BIRC2; EGFR; RELA; EFNB2; DLL4; SDCBP; FRZB; STAT6; STX4; S1PR3; S1PR2; SH2B3; LYN; STAT5A; JUN; XBP1; TGFB2; JUND; NIBAN2; STAT1; TNFRSF9; IL34; STAT3; MEAK7; LIF; OSGIN1; VEGFC; NMI; INHBA; SOD2; MYO16; PML; WARS1; FOSL1; CXCL10; CXCL11; EPGN; IL6; BCL6; BAMBI; TNFSF4; IL7R; PTPN2*
cellular response to tumor necrosis factor (GO:0071356)	*CD40; CXCL8; TNFRSF11B; TANK; TNF; YBX3; CX3CL1; RELA; PSMB10; ZFP36; CCL5; CCL2; GBP2; TNFRSF4; GBP1; NKX3-1; GBP3; TNFSF18; TNFSF14; TNFSF15; CCL20; CD70; STAT1; TNFRSF9; TRAF2; TRAF1; TNFRSF1B; PSMB9; NFKBIA; TRAF3; TNFSF4; LTA; LTB; TP53; CARD14; BIRC2; TNFRSF21; BIRC3*
positive regulation of cytokine production (GO:0001819)	*CD40; SERPINE1; PIK3CD; TNF; IFIH1; CYP1B1; CD34; GBP5; BTN3A1; IL1R1; IL15; RIPK2; DDX58; NFAM1; SPHK1; TRAF2; TICAM1; RUNX1; IL1A; SLC7A5; IL1B; IRF1; IRF7; TLR7; LTB; RAB7B; IL6ST; PTGER4; CD274; CEBPB; EBI3; NOD2; PTGS2; HIF1A; RELA; C3; IFI16; STAT6; CD74; AFAP1L2; XBP1; RFTN1; STAT3; NFKB1; NFKB2; IL6; STING1; TNFSF4; SAA1*

regulation of I-kappaB kinase/NF-kappaB signaling (GO:0043122)	*CD40; GOLT1B; IFIT5; SECTM1; TNFAIP3; DDX21; NOD2; LITAF; TANK; TNF; CX3CL1; RELA; TRIM8; LGALS1; TIFA; TNFSF10; TRIM25; TRIM21; IKBKE; APOL3; CD74; RIPK2; STAT1; PLK2; TNFRSF10B; TRAF2; TRAF1; CFLAR; TICAM1; GPRC5B; TNIP1; TRAF3; TNIP2; IL1B; TNIP3; SQSTM1; OPTN; BIRC2; BIRC3*
cellular response to interferon-gamma (GO:0071346)	*CIITA;CX3CL1;ICAM1;TRIM8;MT2A;CCL5;TRIM25;CCL2;GBP2;TRIM21;GBP1;GBP4;JAK1;GBP3;GBP5;VCAM1;IFNGR1;CCL20;STAT1;IFNGR2;PRKCD;PML;IRF1;OAS3;IRF2;IRF7;CD47;TRIM31*
**Enriched pathways with downregulated genes**	
anterior/posterior pattern specification (GO:0009952)	*HES7; TBX1; HEYL; BMP2; HEY1; LHX1; HOXA3; HOXB3; HOXA6; HOXA5; HOXB6; HOXC6*
generation of neurons (GO:0048699)	*HES7; IRX1; MEF2C; WNT2B; LDB1; FZD10; WNT9A; POU3F2; CIT; HEYL; WNT6; OPHN1; HEY1; MAP1B; LHX1; RAPGEF2; LMX1B; LHX4; ERCC6; WNT2; WNT3; LHX9*
neuron differentiation (GO:0030182	*EPHA5; IRX1; MEF2C; WNT2B; LDB1; FZD10; WNT9A; POU3F2; PBX1; GLI2; WNT6; OPHN1; ID2; LHX1; LMX1B; LHX4; ERCC6; WNT2; WNT3; LHX9*
ameboidal-type cell migration (GO:0001667)	*TBX1; SEMA6C; SEMA7A; SEMA4D; SEMA3D; SEMA6D; SEMA3G; SOX8; AMOTL1; AMOT*
neural crest cell migration (GO:0001755)	*TBX1; SEMA6C; SEMA7A; SEMA4D; SEMA3D; SEMA6D; SEMA3G; SOX8*
negative regulation of axon extension involved in axon guidance (GO:0048843)	*SEMA6C; SEMA7A; SEMA4D; SEMA3D; SEMA6D; SEMA3G*
endocardial cushion development (GO:0003197)	*BMP4; HEYL; BMP2; HEY1; NOG; CRELD1*
regulation of branching involved in ureteric bud morphogenesis (GO:0090189)	*BMP4; LHX1; NOG; TACSTD2; SOX8*
axonogenesis (GO:0007409)	*EPHA5; NLGN3; SPTBN4; SEMA6C; SEMA7A; RAB3A; SEMA4D; BDNF; SEMA3D; PLPPR4; SEMA6D; SEMA3G; NEXN; ANK3; VLDLR; GLI2; OPHN1; MAP1B; MAP1A; KIF5A; SLITRK6; EPHB3*
regulation of axon extension involved in axon guidance (GO:0048841)	*SEMA6C; SEMA7A; SEMA4D; SEMA3D; SEMA6D; SEMA3G*

Supplementary table S5. Top enriched pathways with upregulated or downregulated genes in *H. pylori*-infected in comparison to control cells

**Term**	Genes
**Enriched pathways with upregulated genes**	
regulation of transcription by RNA polymerase II (GO:0006357)	*ATF1; TCERG1; ZNF296; EHF; PID1; SERPINE1; JMJD1C; IKZF2; GABPB1; BACH1; BACH2; TIAL1; IL4I1; MYC; SOX15; ZXDB; NAMPT; DPF3; EPC2; ZNF721; ZNF600; CCNL1; CCN1; JUNB; IER5; IER2; SOX4; DDX17; MEF2A; ZNF282; IFRD1; DKK1; RUNX2; RUNX1; JMJD6; ISL2; HOXB9; DDIT3; TRIB3; PRKD1; HOXB8; ATF3; ATF4; ZNF276; ZNF155; ZNF274; EPAS1; GATA6; ZNF20; ZBTB1; MYNN; NEDD4L; HIF1A; ZNF26; ZBTB2; NHLH2; ZNF829; ZNF827; HIVEP1; HIVEP3; HIVEP2; CGA; LYAR; HOXC8; ZNF267; ZBED4; ZNF146; ZNF266; ZBED3; STAT5A; EGR1; PLK3; JUN; EGR2; XBP1; ZNF384; ZNF263; ZNF142; JAG1; EGR3; JUND; ZNF140; FZD5; GADD45A; LIF; IRF2BP2; NR1D2; NR0B1; PBX4; NR1D1; INO80; FOSL2; SOX30; FOSL1; SNW1; ZNF814; REL; CREBRF; GTF2IRD1; ZNF256; EZH2; ZNF134; ZNF496; CSRNP1; HMX3; CSRNP2; CDKN1A; ZNF131; GMEB2; ZNF250; CITED2; CITED4; AATF; ZNF48; GLI3; PHF8; MED13; MED10; ZMYM5; ANKRD1; ZNF367; ZNF124; TEAD4; IL11; BRD2; ZNF121; ZHX2; NCOA5; RIPK2; TFE3; TFEB; BAZ1A; FOS; BAZ1B; PPRC1; SIRT1; MMP12; PLSCR1; ZEB2; SLTM; NFX1; MTF1; TET3; RARA; AHRR; NCOA7; ARHGEF2; JDP2; ZNF595; ZNF473; FOXC2; EGFR; RELA; RELB; FSTL3; ARNTL; NFIL3; TBL1X; HDX; ZNF343; BRD4; ZNF584; CDKN2B; IFNB1; ATAD2; STAT3; GTF2H1; GRHL1; VEGFA; HIPK2; GATAD2A; PER2; PER1; WT1; FOSB; ZNF699; ZNF697; GZF1; CRTC2; CRTC3; CCNT1; WWC1; ZBTB21; ZBTB20; ARID4B; AHR; ETS1; FGF2; TNF; ETS2; FOXQ1; ZFP36; WWC3; ZNF568; KMT5A; ZNF324; ZNF202; ZBTB39; MSX2; ZBTB38; ZFP69B; ARID5A; HIC1; DMTF1; ZNF317; ZNF316; SQSTM1; ZNF674; ZNF551; ZNF550; DLX2; DDX5; ZNF670; AKNA; MAX; CREM; GLIS3; PSEN1; FOXO3; NPAS1; GLIS2; SBNO2; SAFB2; ZKSCAN2; ZNF787; ZNF302; ZNF300; ZNF783; AKIRIN2; ZBTB17; AKIRIN1; SMAD3; VDR; ZBTB10; INHBA; NFKB1; ESR2; NFKB2; EFNA1; NFKBIA; NR4A2; IL6; CDK7; DLG1; BCL6; MAFG; BHLHE40; MAFF; RFXAP; JMY; ZNF777; MAFK; MXD1; ZNF773; NFE2L2; ZNF770; DDX3X; MIDEAS; AKAP8L; FHL2; CHD2; CHD1; PKD1; FLCN; ZNF408; ZNF529; ZNF768; ZNF525; ZNF644; MYBL1; NKX3-1; KDM6B; KLF10; KLF11; MYOCD; KLF12; TGIF2; BUD31; VEZF1; OLIG2; SAFB; ETV4; KLF15; ETV5; ZNF75A; KLF16; ETV6; SARS1; IRF1; IRF2; CRY1; IRF7; ZNF875; KDM7A; CREB5; NFAT5; CEBPB; CEBPD; CEBPG; ZBTB48; ZBTB43; NR2C2; HDAC9; ATAD2B; DLL4; IFI16; ZNF749; E2F1; RBBP8; E2F2; E2F3; HES1; ZNF501; ZNF621; FNIP1; ZNF620; E2F7; HES4; SPEN; KDM4C; CNBP; SOD2; KLF4; KLF3; TBX3; TBX2; KLF6; KLF5; TNIP1; TNIP2; TNIP3; DNAJA3; KLF9; CEBPZ; BCOR; PPIL4; TAF1*
regulation of transcription, DNA-templated (GO:0006355)	*ATF1; ZNF296; EHF; JMJD1C; IKZF2; BACH1; BACH2; TIAL1; MYC; SOX15; ZXDB; GPBP1; DPF3; EPC2; FBXO5; ZNF721; ZNF600; CCNL1; JUNB; SOX4; DDX17; MEF2A; ZNF282; SFMBT2; IFRD1; RUNX2; RUNX1; JMJD6; ISL2; ZNF718; HOXB9; DDIT3; TRIB3; HOXB8; ATF3; ATF4; ZNF276; ZNF155; ZNF274; EPAS1; IGSF1; GATA6; ZNF20; ZBTB1; MYNN; AKAP17A; HIF1A; ZNF26; ZBTB2; NHLH2; ZNF829; HIVEP1; HIVEP3; HIVEP2; CGA; HOXC8; ZNF267; ZNF146; ZNF266; STAT5A; CDK17; EGR1; JUN; EGR2; PHC2; ZNF384; AFAP1L2; ZNF263; ZNF142; EGR3; JUND; ZNF140; IRF2BP2; NR1D2; NR0B1; PBX4; NR1D1; FOSL2; SOX30; FOSL1; SNW1; ZNF814; TNFSF4; REL; CREBRF; GTF2IRD1; ZNF256; EZH2; ZNF134; ZNF496; CSRNP1; SETD5; HMX3; CSRNP2; CDKN1A; ZNF131; GMEB2; BTG1; ZNF250; CITED2; PHF1; AATF; ZNF48; GLI3; PHF8; ING5; MED13; SCML1; UIMC1; ZNF280C; ANKRD1; ZNF367; ZNF124; TEAD4; BRD2; ZNF121; ZHX2; TFE3; TFEB; BAZ1A; FOS; BAZ1B; RGMB; SIRT1; ZEB2; SLTM; NFX1; IL1B; MTF1; RARA; WAC; AHRR; NCOA7; JDP2; ZNF595; ZNF473; FOXC2; EGFR; RELA; RELB; FSTL3; ARNTL; BTAF1; NFIL3; METTL4; HAS3; APBB2; TBL1X; HDX; ZNF343; BRD4; ZNF584; UTP4; ATAD2; STAT3; GTF2H1; WTIP; GRHL1; VEGFA; HIPK2; GATAD2A; PER2; PER1; RYBP; WT1; FOSB; ZNF699; ZNF697; GZF1; CCNT1; NAB1; WWC1; ZBTB21; ZBTB20; ARID4B; AHR; ETS1; FGF2; TNF; ETS2; FOXQ1; WWC3; ZNF568; KMT5A; ZNF324; ZNF202; MAP2K3; ZBTB39; MSX2; ZBTB38; ZFP69B; ARID5A; HIC1; DMTF1; CCNE1; ZNF317; ZNF316; AJUBA; ZNF674; ZNF551; ZNF550; DLX2; DDX5; ZNF670; MAX; CREM; GLIS3; PSEN1; FOXO3; NPAS1; GLIS2; SBNO2; SAFB2; ZKSCAN2; ZNF787; ZNF302; ZNF300; ZNF783; ZBTB17; BRPF1; SMAD3; DENND4A; VDR; ZBTB10; INHBA; FOXN2; NFKB1; ESR2; NFKB2; NR4A2; IL6; BCL6; MAFG; BHLHE40; BCL3; MAFF; RFXAP; JMY; ZNF777; MAFK; MXD1; ZNF773; NFE2L2; ZNF770; MIDEAS; ZC3H8; CHD2; CHD1; ZNF408; ZNF529; ZNF768; ZNF525;*

	ZNF644; YAF2; MYBL1; NKX3-1; KLF10; KLF11; MYOCD; KLF12; TGIF2; RBM15; DTX3L; AXIN1; NRG1; VEZF1; OLIG2; GLRX2; SAFB; ETV4; KLF15; ETV5; ZNF75A; REXO4; KLF16; ETV6; DNAJC2; IRF1; IRF2; CRY1; IRF7; ZNF875; KDM7A; CREB5; NFAT5; CEBPB; CEBPD; ZNF195; CEBPG; ZBTB48; ZBTB43; RNF6; NR2C2; HDAC9; IFNL1; IFI16; CCDC85B; ZNF749; TP53INP2; ZNF506; E2F1; RBBP8; E2F2; E2F3; HES1; ZNF502; ZNF501; ZNF621; ZNF620; E2F7; HES4; SPEN; KDM4C; CNBP; SOD2; KLF4; YY1AP1; KLF3; TBX3; TBX2; KLF6; KLF5; TCIM; POLR3C; KLF9; POLR3F; BCOR
positive regulation of transcription by RNA polymerase II (GO:0045944)	*ATF1; TCERG1; ZNF296; CRTC2; EHF; PID1; CRTC3; CCNT1; WWC1; SERPINE1; AHR; GABPB1; ETS1; FGF2; TNF; IL4I1; MYC; SOX15; NAMPT; DPF3; CCN1; IER5; JUNB; IER2; SOX4; MEF2A; DDX17; ZBTB38; RUNX2; JMJD6; RUNX1; DDIT3; PRKD1; SQSTM1; ATF3; ATF4; AKNA; MAX; EPAS1; GATA6; GLIS3; FOXO3; HIF1A; GLIS2; NHLH2; SBNO2; ZNF827; HIVEP1; CGA; ZBED4; ZNF300; ZBED3; ZBTB17; EGR1; AKIRIN2; JUN; ZNF263; EGR2; XBP1; AKIRIN1; TGFB2; JAG1; JUND; SMAD3; FZD5; VDR; LIF; NR1D2; INHBA; INO80; NR1D1; NFKB1; NFKB2; FOSL2; SOX30; FOSL1; NFKBIA; NR4A2; IL6; CDK7; SNW1; MAFF; RFXAP; REL; CREBRF; NFE2L2; CSRNP1; CSRNP2; DDX3X; CITED2; AKAP8L; CITED4; AATF; PKD1; GLI3; FLCN; MED13; MED10; MYBL1; NKX3-1; KDM6B; KLF10; IL11; MYOCD; RIPK2; TFE3; TFEB; VEZF1; FOS; PPRC1; SIRT1; ETV4; KLF15; ETV5; GNL3; MMP12; PLSCR1; ZEB2; IRF1; MTF1; IRF2; TET3; IRF7; RARA; NCOA7; ARHGEF2; FOXC2; NFAT5; CEBPB; CEBPG; NR2C2; EGFR; RELA; FSTL3; ATAD2B; ARNTL; IFI16; E2F1; E2F2; E2F3; HES1; TBL1X; E2F7; BRD4; CDKN2B; IFNB1; CNBP; ATAD2; STAT3; KLF4; GRHL1; TBX3; VEGFA; HIPK2; PER1; KLF6; KLF5; TNIP1; WT1; TNIP2; TNIP3; CEBPZ*
positive regulation of transcription, DNA-templated (GO:0045893)	*ATF1; TCERG1; ZNF296; EHF; PID1; SERPINE1; GABPB1; IL4I1; MYC; SOX15; NAMPT; DPF3; CCN1; IER5; JUNB; IER2; SOX4; MEF2A; DDX17; RUNX2; JMJD6; RUNX1; DDX39B; DDIT3; PRKD1; ATF3; ATF4; EPAS1; GATA6; HIF1A; NHLH2; ZNF827; HIVEP1; HIVEP3; CGA; LYAR; ZBED4; ZBED3; EGR1; JUN; ZNF263; EGR2; XBP1; AFAP1L2; JAG1; JUND; FZD5; EIF2AK3; LIF; NR1D2; PBX4; INO80; NR1D1; FOSL2; SOX30; FOSL1; SNW1; REL; CREBRF; ZNF496; CSRNP1; CSRNP2; CITED2; CITED4; AATF; GLI3; WDR43; PHF8; ING5; MED13; MED10; IL11; RIPK2; TFE3; TFEB; FOS; RGMB; PPRC1; SIRT1; MMP12; PLSCR1; ZEB2; IL1B; MTF1; TET3; RARA; WAC; NCOA7; ARHGEF2; FOXC2; EGFR; RELA; FSTL3; ARNTL; NFIL3; SERTAD1; SERTAD2; HAS3; TBL1X; BRD4; CDKN2B; IFNB1; ATAD2; STAT3; GTF2H1; GRHL1; VEGFA; HIPK2; PER1; RYBP; WT1; CRTC2; CRTC3; CCNT1; AHR; ETS1; FGF2; TNF; ETS2; MAP2K3; ZBTB38; SQSTM1; AKNA; MAX; GLIS3; PSEN1; FOXO3; GLIS2; SBNO2; ZNF300; ZBTB17; AKIRIN2; AKIRIN1; BRPF1; SMAD3; VDR; INHBA; NFKB1; ESR2; NFKB2; NFKBIA; NR4A2; IL6; CDK7; BCL3; MAFF; RFXAP; NFE2L2; DDX3X; AKAP8L; ZC3H8; PKD1; FLCN; YAF2; MYBL1; NKX3-1; UTP15; KDM6B; KLF10; MYOCD; DTX3L; AXIN1; VEZF1; ETV4; KLF15; ETV5; DNAJC2; IRF1; IRF2; IRF7; CREB5; NFAT5; CEBPB; ZBTB48; CEBPG; RNF6; NR2C2; ATAD2B; IFNL1; IFI16; TP53INP2; E2F1; E2F2; E2F3; HES1; E2F7; CNBP; KLF4; TBX3; KLF6; KLF5; TNIP1; TNIP2; TNIP3; CEBPZ*
negative regulation of transcription by RNA polymerase II (GO:0000122)	*TCERG1; WWC1; ZBTB21; BACH1; TNF; ETS2; ZFP36; MYC; SOX15; WWC3; ZNF568; KMT5A; ZNF202; MEF2A; MSX2; ZNF282; ZFP69B; ARID5A; DKK1; HIC1; RUNX1; DDIT3; TRIB3; ATF3; ZNF674; ATF4; ZNF550; ZNF274; DLX2; DDX5; MAX; GATA6; ZNF20; CREM; GLIS3; ZBTB1; NEDD4L; PSEN1; FOXO3; GLIS2; ZBTB2; HIVEP1; LYAR; ZNF266; PLK3; JUN; ZNF263; SMAD3; ZNF140; GADD45A; VDR; IRF2BP2; NR0B1; NR1D2; NR1D1; NFKB1; ESR2; SOX30; EFNA1; NR4A2; DLG1; SNW1; BCL6; BHLHE40; GTF2IRD1; MAFK; MXD1; EZH2; CITED2; FHL2; GLI3; FLCN; ZNF529; ZMYM5; ZNF124; KLF10; KLF11; ZHX2; KLF12; TGIF2; NCOA5; SIRT1; ZNF75A; ETV5; ETV6; ZEB2; SARS1; NFX1; IRF2; RARA; CRY1; IRF7; ZNF875; JDP2; FOXC2; CEBPB; NR2C2; HDAC9; RELA; DLL4; IFI16; NFIL3; ZNF749; E2F1; HES1; TBL1X; FNIP1; ZNF620; E2F7; ZNF343; SPEN; CNBP; STAT3; KLF4; TBX3; GATAD2A; TBX2; VEGFA; PER2; PER1; WT1; DNAJA3; NEDD4; FOSB; BCOR; GZF1; TAF1*
negative regulation of transcription, DNA-templated (GO:0045892)	*TCERG1; NAB1; WWC1; ZBTB21; AHR; BACH1; TNF; ETS2; ZFP36; MYC; SOX15; WWC3; DPF3; ZNF568; KMT5A; ZNF202; MEF2A; MSX2; ZNF282; ZBTB38; SFMBT2; ZFP69B; ARID5A; DKK1; HIC1; RUNX2; RUNX1; DDIT3; TRIB3; ATF3; ZNF674; ATF4; ZNF550; ZNF274; DLX2; DDX5; MAX; GATA6; ZNF20; CREM; GLIS3; ZBTB1; NEDD4L; PSEN1; FOXO3; NPAS1; GLIS2; ZBTB2; SBNO2; HIVEP1; LYAR; ZNF266;*

	PLK3; JUN; ZNF263; PHC2; SMAD3; ZNF140; VDR; GADD45A; IRF2BP2; NR0B1; NR1D2; NR1D1; NFKB1; ESR2; SOX30; EFNA1; NR4A2; DLG1; SNW1; BCL6; TNFSF4; BHLHE40; BCL3; GTF2IRD1; MAFK; MXD1; ZNF256; EZH2; CITED2; MIDEAS; FHL2; ZC3H8; GLI3; FLCN; SCML1; ZNF529; ZMYM5; UIMC1; YAF2; ZNF124; NKX3-1; KLF10; KLF11; ZHX2; KLF12; RBM15; TGIF2; NCOA5; NRG1; SIRT1; ETV5; ZNF75A; ETV6; ZEB2; SARS1; NFX1; IRF1; IRF2; CRY1; IRF7; RARA; ZNF875; JDP2; FOXC2; CEBPB; NR2C2; HDAC9; RELA; ARNTL; DLL4; BTAF1; IFNL1; IFI16; NFIL3; CCDC85B; ZNF749; E2F1; HES1; TBL1X; FNIP1; ZNF620; E2F7; ZNF343; SPEN; CNBP; STAT3; KLF4; TBX3; GATAD2A; TBX2; VEGFA; PER2; PER1; WT1; DNAJA3; FOSB; BCOR; GZF1; TAF1
cytokine-mediated signaling pathway (GO:0019221)	*CNTF; CSF1; IRS1; IL1RAP; FGF2; TNF; ICAM1; MT2A; MYC; GRB10; TRIM25; JUNB; IL6R; IL15RA; IL4R; GPR75; MTAP; ADAM17; IL6ST; SQSTM1; STX1A; IL20RB; FOXO3; HIF1A; ADIPOR2; SOCS2; SOCS3; UGCG; HNRNPDL; IRAK2; ABL2; MCL1; STAT5A; TNFSF18; HSPA9; EGR1; VCAM1; TNFSF14; TNFSF15; TNFRSF9; LIF; NFKB1; NFKBIA; FER; IL6; BCL6; HNRNPF; TNFSF4; IL7R; MAP3K14; CXCL6; CDKN1A; ITGAM; CXCL8; IL24; CXCL1; CXCL3; CXCL2; IL11; SERPINB2; TNFRSF12A; GPR35; IFNGR1; RIPK2; IL15; MMP1; IFNGR2; TRAF2; FOS; TRAF1; OSMR; F3; SIRT1; IL1A; CEACAM1; TRAF3; IL1B; IRF1; IL3RA; IRF2; PELI1; IRF7; LTA; LCP1; LTB; BIRC2; BIRC3; CEBPD; PTGS2; IL2RG; RELA; IL1RL1; IFNL1; IL21R; CCL2; STX3; SH2B3; GBP1; IL32; CCL20; CD70; IFNB1; IL34; STAT3; SOD2; VEGFA; TNIP2; SAA1; TRIM31*
cellular response to cytokine stimulus (GO:0071345)	*GSK3B; CNTF; CDKN1A; PID1; ITGAM; CXCL8; CSF1; IL24; MT1X; CXCL1; IL1RAP; FGF2; TNF; CXCL2; ICAM1; ZFP36; MT2A; MYC; GRB10; ANKRD1; JUNB; IL6R; NKX3-1; IL11; IL4R; DUSP1; IFNGR1; MMP1; IFNGR2; FOS; OSMR; F3; SIRT1; IL1A; IL1B; IRF1; IL3RA; ARHGEF2; IL6ST; TNFRSF21; STX1A; NFAT5; SELPLG; CEBPD; IL20RB; CXCR4; PTGS2; FOXO3; IL2RG; HIF1A; TANK; RELA; ZFP36L2; ZFP36L1; SOCS2; SOCS3; SBNO2; IFNL1; IFI16; IRAK2; ABL2; CCL2; STX3; SH2B3; GBP1; MCL1; GBP3; STAT5A; IL32; EGR1; XBP1; SMAD3; VCAM1; CCL20; IFNB1; IL34; STAT3; LIF; ERBIN; NR1D1; NFKB1; VEGFA; FER; IL6; LRCH1; BCL6; SAA1; PTPN2; NFE2L2*
regulation of gene expression (GO:0010468)	*MYLK2; CNTF; PID1; CSF1; NAB1; WWC1; JMJD1C; AHR; ETS1; BACH1; FGF2; TNF; FGF5; CDH5; MYC; SOX15; GPBP1; WWC3; ICOSLG; SOX4; MBNL2; ZNF282; ZBTB38; SFMBT2; TICAM1; ATP1B1; PRKAB1; DKK1; HIC1; RUNX1; OLFM1; ZNF718; DMTF1; DDIT3; ALPK2; AJUBA; ATF4; ZNF274; YTHDC1; IGSF1; CREM; PSEN1; AKAP17A; MEFV; HIF1A; ZFP36L1; SBNO2; HNRNPDL; PCBP3; ZNF827; STC2; SLC38A2; FGF22; ZNF146; FGF21; EGR1; CPSF4; ZNF263; TGFB2; SMAD3; BTN2A2; TIPARP; DENND4A; VDR; BTN2A1; EIF2AK3; RAB27A; BRAF; NR1D2; INHBA; LARP4; FOXN2; CLK4; NFKB1; ESR2; NFKB2; CLK1; IL6; CDK6; BCL6; HNRNPF; BHLHE40; REL; GTF2IRD1; EZH2; NFE2L2; GSK3B; USP36; DDX3X; BTG1; CXCL8; CELF1; CITED2; PHF1; AFF3; AFF4; FLCN; C1QTNF1; TRIM4; ZNF280C; ITGB8; AMH; YAF2; MYBL1; NKX3-1; KDM6B; TGIF2; IFNGR1; TFEB; SERPINB9; GLRX2; NGF; F3; SIRT1; REXO4; SLC7A5; IL1A; PLSCR1; TRAF3; NFX1; PTRH2; IL1B; IRF2; TRIM58; ZNF875; CEBPB; ZNF195; RNF6; NR2C2; RELA; PTHLH; ARNTL; DLL4; IFI16; NFIL3; TDG; NOCT; ZNF506; E2F1; METTL4; ZNF502; APBB2; IGF2BP2; TRIM40; UTP4; CNBP; STAT3; WTIP; KLF4; YY1AP1; WARS1; VEGFA; PER1; RYBP; TRIM39; TRIML2; WT1; ADM2; SPRY2; TRIM31; GZF1; TAF1*
positive regulation of pri-miRNA transcription by RNA polymerase II (GO:1902895)	*EGR1; JUN; TGFB2; SMAD3; STAT3; FOS; FOXO3; KLF4; ETS1; FGF2; HIF1A; TNF; RELA; GNL3; FOSL1; KLF5; WT1*
**Enriched pathways with downregulated genes**	
chaperone cofactor-dependent protein refolding (GO:0051085)	*DNAJB1; HSPA8; SDF2L1; HSPA1L; HSPA5; DNAJB5; DNAJB4; HSPA6; HSPA2; HSPA1B; HSPA1A*
'de novo' posttranslational protein folding (GO:0051084)	*DNAJB1; HSPA8; SDF2L1; HSPA1L; HSPA5; DNAJB5; DNAJB4; HSPA6; HSPA2; HSPA1B; HSPA1A*

response to unfolded protein (GO:0006986)	*HSPA8; HSPA1L; HSPA5; HSPB7; HSPA6; HSPB1; HSPA2; DNAJB1; BAG3; DNAJB5; DNAJB4; HSPA1B; HSPA1A*
cellular response to unfolded protein (GO:0034620)	*HSPA8; HSPA1L; HSPA5; BAG3; HSPA6; HSPA2; HSPA1B; HSPA1A*
cellular response to steroid hormone stimulus (GO:0071383)	*BMP4; AR; GPER1; OR51E2; AKR1C3; MGARP; TRERF1*
aminoglycan biosynthetic process (GO:0006023)	*B4GAT1; B3GALT2; SDC3; XYLT2; B3GNT3; GCNT2; PXYLP1; SDC1; AGRN; HS6ST1; HSPG2*
embryonic organ morphogenesis (GO:0048562)	*TBX1; MESP1; OSR2; RARG; FOXE1; ID2; HOXA3; HOXB4; TCF21; MEGF8*
negative regulation of viral process (GO:0048525)	*APOBEC3C; HEXIM1; TRIM62; RNASEL; APOBEC3F; EIF2AK2; IFIT1; TRIM6; OAS1; TRIM5; OAS2; TRIM14; TRIM21*
regulation of cyclin-dependent protein kinase activity (GO:1904029)	*CDKN1C; CCNA2; CCNB1; CDKN2C; CCNG2; CCNI2; CDC25C; CCNP; CDK5RAP3; CDK5R2; CCNJL*
odontogenesis (GO:0042476)	*TBX1; BMP4; WNT6; OSR2; EDA; COL1A2; AXIN2; PITX2; GLI2*

Supplementary table S6. Top common enriched pathways with upregulated or downregulated genes in TNF-treated and *H. pylori*-infected cells in comparison to untreated cells

Term	overlap	p value	Adjusted p value	Genes
**Enriched pathways with upregulated geness**				
cytokine-mediated signaling pathway (GO:0019221)	72	$1.25x10^{-27}$	$3.60x10^{-24}$	*CXCL6; CDKN1A; ITGAM; CXCL8; CSF1; CXCL1; CXCL3; TNF; CXCL2; ICAM1; MT2A; TRIM25; JUNB; IL15RA; SERPINB2; IL4R; RIPK2; IFNGR1; IL15; IFNGR2; TRAF2; TRAF1; OSMR; IL1A; CEACAM1; TRAF3; IL1B; IRF1; IL3RA; IRF2; LTA; IRF7; LCP1; LTB; IL6ST; SQSTM1; BIRC2; BIRC3; CEBPD; PTGS2; IL2RG; HIF1A; RELA; SOCS3; UGCG; IRAK2; CCL2; SH2B3; GBP1; MCL1; TNFSF18; STAT5A; IL32; VCAM1; TNFSF14; CCL20; TNFSF15; CD70; TNFRSF9; IL34; STAT3; LIF; SOD2; NFKB1; NFKBIA; IL6; BCL6; TNIP2; TNFSF4; SAA1; TRIM31; IL7R*
cellular response to cytokine stimulus (GO:0071345)	53	$1.95x10^{-19}$	$2.82x10^{-16}$	*CDKN1A; ITGAM; CXCL8; CSF1; MT1X; CXCL1; TNF; CXCL2; ICAM1; ZFP36; MT2A; JUNB; NKX3-1; IL4R; IFNGR1; IFNGR2; OSMR; IL1A; IL1B; IRF1; IL3RA; IL6ST; TNFRSF21; SELPLG; CEBPD; PTGS2; IL2RG; HIF1A; TANK; RELA; SOCS3; SBNO2; IFI16; IRAK2; CCL2; SH2B3; GBP1; MCL1; GBP3; STAT5A; IL32; XBP1; SMAD3; VCAM1; CCL20; IL34; STAT3; LIF; NFKB1; IL6; BCL6; SAA1; PTPN2*
cellular response to lipopolysaccharide (GO:0071222)	24	$3.44x10^{-15}$	$3.31x10^{-12}$	*CD274; CXCL6; XBP1; CXCL8; SERPINE1; TNFAIP3; CXCL1; PDCD1LG2; TICAM1; CXCL3; TNF; CXCL2; NFKB1; RELA; IL1A; ZFP36; SBNO2; IL6; TNIP2; TNFSF4; IL1B; TNIP3; CCL2; NFKBIB*
cellular response to molecule of bacterial origin (GO:0071219)	23	$1.30x10^{-14}$	$9.41x10^{-12}$	*CD274; CXCL6; XBP1; CXCL8; SERPINE1; TNFAIP3; CXCL1; PDCD1LG2; TICAM1; CXCL3; CXCL2; NFKB1; RELA; IL1A; ZFP36; SBNO2; IL6; TNIP2; TNFSF4; IL1B; TNIP3; CCL2; NFKBIB*
response to lipopolysaccharide (GO:0032496)	26	$3.82x10^{-14}$	$2.21x10^{-11}$	*CD274; CXCL6; CXCL8; SERPINE1; TNFAIP3; CXCL1; CXCL3; CXCL2; RELA; ZFP36; SBNO2; NOCT; CCL2; XBP1; GCH1; PDCD1LG2; TICAM1; NFKB1; IL1A; IL6; TNIP2; TNFSF4; IL1B; TNIP3; TRIB1; NFKBIB*
regulation of inflammatory response (GO:0050727)	29	$7.01x10^{-14}$	$3.38x10^{-11}$	*PTGER4; SERPINE1; TNFAIP3; MEFV; PTGS2; ETS1; TNF; RELA; CDH5; SBNO2; CASP4; IL15; SPHK1; NFKBIA; CYLD; MFHAS1; GPRC5B; IL6; BCL6; TNIP1; TNFSF4; IL1B; NINJ1; SAA1; TLR7; BIRC2; PTPN2; PTGES; BIRC3*
positive regulation of transcription by RNA polymerase II (GO:0045944)	62	$1.27x10^{-12}$	$5.24x10^{-10}$	*CSRNP1; EHF; ZNF296; WWC1; SERPINE1; CITED4; AHR; GABPB1; TNF; ETS1; GLI3; IL4I1; NAMPT; JUNB; IER5; MYBL1; NKX3-1; KDM6B; KLF10; RIPK2; TFE3; ETV4; RUNX1; PLSCR1; IRF1; IRF2; IRF7; NCOA7; SQSTM1; ATF3; FOXC2; CEBPB; MAX; EPAS1; HIF1A; EGFR; RELA; FSTL3; SBNO2; IFI16; HIVEP1; JUN; XBP1; TGFB2; JUND; SMAD3; STAT3; LIF; INHBA; NFKB1; FOSL2; NFKB2; FOSL1; NFKBIA; NR4A2; KLF6; IL6; KLF5; TNIP1; TNIP2; TNIP3; MAFF*
positive regulation of cytokine production (GO:0001819)	35	$1.50x10^{-12}$	$5.41x10^{-10}$	*PTGER4; CD274; CEBPB; SERPINE1; PTGS2; HIF1A; TNF; RELA; IFIH1; IFI16; CYP1B1; AFAP1L2; XBP1; IL15; RIPK2; SPHK1; RFTN1; STAT3; TRAF2; TICAM1; NFKB1; RUNX1; NFKB2; IL1A; SLC7A5; IL6; TNFSF4; IL1B; IRF1; SAA1; IRF7; TLR7; LTB; RAB7B; IL6ST*
inflammatory response (GO:0006954)	28	$7.10x10^{-12}$	$2.28x10^{-9}$	*PTGER4; CXCL6; CEBPB; CXCL8; CXCL1; MEFV; CXCL3; TNF; CXCL2; HDAC9; RELA; RELB; HRH1; OLR1; CCL2; AFAP1L2; RIPK2; CCL20; STAT3; PLA2G4C; NFKB1; IL1A; MFHAS1; IL6; KRT16; TNFSF4; IL1B; PTX3*

positive regulation of transcription, DNA-templated (GO:0045893)	71	1.16×10^{-11}	3.30×10^{-9}	*CSRNP1; EHF; ZNF296; SERPINE1; CITED4; AHR; GABPB1; TNF; ETS1; GLI3; ETS2; IL4I1; NAMPT; JUNB; IER5; YAF2; MYBL1; NKX3-1; MAP2K3; KDM6B; KLF10; DTX3L; RIPK2; TFE3; ETV4; RUNX1; PLSCR1; IL1B; IRF1; IRF2; IRF7; NCOA7; SQSTM1; ATF3; FOXC2; CEBPB; MAX; EPAS1; PSEN1; HIF1A; EGFR; RELA; FSTL3; SBNO2; IFI16; NFIL3; HIVEP1; HIVEP3; JUN; AFAP1L2; XBP1; JUND; SMAD3; STAT3; LIF; PBX4; INHBA; NFKB1; FOSL2; NFKB2; FOSL1; NFKBIA; NR4A2; KLF6; IL6; KLF5; TNIP1; TNIP2; TNIP3; MAFF; BCL3*
**Enriched pathways with downregulated genes**				
cellular response to steroid hormone stimulus (GO:0071383)	5	1.83×10^{-5}	2.09×10^{-2}	*BMP4; GPER1; OR51E2; MGARP; TRERF1*
neuron differentiation (GO:0030182)	12	2.97×10^{-5}	2.09×10^{-2}	*IRX1; WNT6; MEF2C; LDB1; ID2; LHX1; LMX1B; POU3F2; WNT2; PBX1; LHX9; GLI2*
odontogenesis (GO:0042476)	6	3.71×10^{-5}	2.09×10^{-2}	*TBX1; BMP4; WNT6; OSR2; EDA; GLI2*
anterior/posterior pattern specification (GO:0009952)	7	7.25×10^{-5}	3.06×10^{-2}	*HES7; TBX1; LHX1; HOXA3; HOXA6; HOXA5; HOXB6*
axon development (GO:0061564)	7	3.04×10^{-4}	8.56×10^{-2}	*SPTBN4; RAB3A; AMIGO1; MAP1A; SLITRK6; MAPK8IP3; EPHB3*
regulation of transcription by RNA polymerase II (GO:0006357)	56	5.15×10^{-4}	1.20×10^{-1}	*LDB1; THRA; GLI2; DMBX1; DBP; ZFP30; HNF4A; GPER1; HOXA3; HOXA6; HOXA5; HES7; BATF2; OSR2; MEF2C; ZNF362; MAMSTR; DNMT3A; TET1; ETV1; POU3F2; FOXP2; RGMA; MYCL; KIT; HOXB2; MAML3; LMX1B; ZNF556; HOXB6; ZNF397; DLX3; TXK; FOXO4; HOXB13; ZKSCAN3; ZNF821; ZNF467; WNT2; CBX8; ZBTB18; TBX1; IRX1; STAT2; PTCH1; ARID3A; TRERF1; ZFHX4; FOXN1; PBX1; BMP4; ID2; LHX1; CAPRIN2; MXD3; LHX9*
cell morphogenesis involved in neuron differentiation (GO:0048667)	6	1.50×10^{-3}	1.67×10^{-1}	*SPTBN4; MEF2C; RAB3A; MAP1A; SLITRK6; MAP6*
ameboidal-type cell migration (GO:0001667)	5	1.55×10^{-3}	1.67×10^{-1}	*TBX1; SEMA6C; SEMA7A; SEMA6D; AMOT*
regulation of cell population proliferation (GO:0042127)	24	1.74×10^{-3}	1.67×10^{-1}	*TBX1; OSR2; CDKN2C; CHRM1; STAT2; RTKN2; NPR3; TET1; FOXO4; SSTR1; POU3F2; SSTR5; BMP4; GRK5; ERBB3; ID2; HNF4A; GPER1; KIT; FGF18; TP53INP1; PTPN6; WNT2; TNS2*
generation of neurons (GO:0048699)	10	1.83×10^{-3}	1.67×10^{-1}	*HES7; IRX1; WNT6; MEF2C; LDB1; LHX1; LMX1B; POU3F2; WNT2; LHX9*

Supplementary table S7. Top enriched pathways with upregulated or downregulated genes in *H. pylori*-infected in comparison to TNF-treated cells

Term	Genes
**Enriched pathways with upregulated genes**	
regulation of transcription, DNA-templated (GO:0006355)	*ATF1; JMJD1C; BACH1; BACH2; CCAR1; TIAL1; RPS6KA5; MYC; SOX15; ZXDC; EPC1; EPC2; SOX8; ZNF721; CCNL1; SAMD11; MEF2A; SFMBT2; IFRD1; RFX3; RUNX2; RUNX1; ISL2; ZNF718; HOXB9; DDIT3; ZSCAN22; ZSCAN21; ZNF713; ZSCAN23; HOXB3; TRIB3; HOXB8; ATF3; ATF4; ZSCAN29; ZNF276; ZNF395; ZNF274; L3MBTL3; SP140; ZNF20; GATA6; XPC; ZNF23; BAZ2B; AKAP17A; ZNF26; MBTD1; NHLH2; ZNF829; HIVEP1; ZNF700; HIVEP3; HIVEP2; ZBED6; PPARGC1A; ZNF146; ZNF266; HOXC6; EGR1; ZNF264; JUN; CREBBP; EGR2; ZNF384; ZNF263; ZNF142; EGR3; FZD4; SS18L1; SETDB2; PBX3; POU6F2; NR1D2; NR1D1; PHC3; MED13L; FOSL2; SOX30; FOSL1; SNW1; ZNF37A; ZNF814; SNAI1; REL; CREBRF; SNAI2; GTF2IRD1; ZNF256; EZH2; ZNF134; ZNF496; LIN54; CSRNP1; SETD5; HMX3; CDKN1A; GMEB2; BTG1; ZNF492; BRCA2; AATF; MED18; ZNF48; ING5; NIPBL; MED13; SCML1; HEY1; ZNF280B; UIMC1; ZNF280C; ANKRD1; ZNF367; TEAD1; ZNF485; ZNF121; BRD2; ZHX2; NCOA6; TFEB; FOS; BAZ1A; BAZ1B; SIRT1; PHF20L1; TOX2; SLTM; IL1B; KIT; RARA; WAC; AHRR; ZNF117; ZNF236; JDP2; ZNF596; ZNF595; ZNF473; ZNF594; ZNF8; BTAF1; NFIL3; METTL4; HAS3; HSF4; MEF2D; ZNF585A; ZNF343; MAP3K2; BRF2; ATAD2; WTIP; GRHL1; VEGFA; PER2; PER1; RYBP; NFIB; FOSB; ZNF699; ZNF578; ZNF577; ZNF697; GZF1; CDK5R1; CCNT1; ZBTB21; ZBTB20; ARID4B; AHR; FGF2; ETS2; FOXQ1; ZNF84; WWC3; ZNF568; KMT5A; ZNF324; ZNF202; ZNF562; ZFP69B; ARID5A; ZBTB34; MITF; HIC1; LBHD1; ZNF91; DMTF1; ZNF93; ZNF317; ZNF316; ZNF678; ZNF557; AJUBA; ZNF674; ZNF430; ZNF551; KMT2D; ZNF550; DLX2; KMT2A; KMT2B; GLIS3; FOXO3; ZNF429; SAFB2; ZKSCAN2; ZNF425; ZNF302; ZNF543; ZNF300; ZNF783; BPTF; TFAP2A; TRAPPC2; ZFHX3; BRPF1; TFAP2E; DENND4A; SMAD9; INHBA; ESR2; SMAD7; NR4A1; NR4A3; SP2; BCL6; SP4; MAFG; MAFF; RFXAP; JMY; PHF14; ZNF778; MAP3K10; MDM4; MAFK; LHX4; MXD1; ZNF773; NFE2L1; NFE2L2; ZNF770; MIDEAS; HMGB2; ZC3H8; CHD2; CHD1; LBH; ZNF408; ZNF528; ZNF768; ZNF644; CEP290; ARHGEF11; KLF10; KLF11; MYOCD; TGIF2; RBM15; ATRX; AXIN1; OLIG2; SAFB; ARID1A; KLF15; ETV5; ZNF75A; REXO4; DNAJC2; CRY1; ZNF875; CREB5; NFAT5; CEBPB; ZNF195; CEBPG; ZBTB48; ZBTB43; RNF6; NR2C2; HDAC9; IFNL1; ZNF749; TP53INP2; ZNF506; E2F1; E2F2; E2F3; HES1; ZNF502; ZNF501; ZNF621; ZNF620; E2F7; E2F8; SPEN; TCF7L2; KDM4A; KDM4C; KLF4; TBX3; KLF6; KLF5; TCIM; ZGLP1; POLR3C; ZNF736; KLF9; PDCD4; NAA16; BCOR; ZNF852; SSBP2*
regulation of transcription by RNA polymerase II (GO:0006357)	*ATF1; PID1; GABPB2; JMJD1C; BACH1; BACH2; TIAL1; RPS6KA5; MYC; SOX15; ZXDC; EPC1; EPC2; SOX8; ZNF721; CCNL1; IER2; MEF2A; IFRD1; RFX3; DKK1; RUNX2; RUNX1; ISL2; HOXB9; DDIT3; ZSCAN22; ZSCAN21; ZSCAN23; HOXB3; TRIB3; PRKD1; HOXB8; ATF3; ATF4; ZSCAN29; ZNF276; ZNF395; ZNF274; SP140; ZNF20; GATA6; NEDD4L; ZNF23; BAZ2B; PRDM10; ZNF26; NHLH2; ZNF829; ZNF827; HIVEP1; ZNF700; HIVEP3; HIVEP2; ZBED6; PPARGC1A; ZNF146; ZNF266; ZBED3; HOXC6; EGR1; ZNF264; JUN; CREBBP; EGR2; XBP1; ZNF384; ZNF263; ZNF142; JAG1; EGR3; GADD45A; SS18L1; PBX3; POU6F2; LIF; LARP7; NR1D2; NR1D1; INO80; H1-0; MED13L; FOSL2; SOX30; FOSL1; SNW1; ZNF37A; ZNF814; SNAI1; REL; CREBRF; SNAI2; GTF2IRD1; CPEB3; ZNF256; EZH2; ZNF134; ZNF496; CSRNP1; HMX3; CDKN1A; GMEB2; AATF; MED18; ZNF48; NIPBL; MED13; HEY1; MED10; ZMYM5; ANKRD1; ZNF367; TEAD1; ZNF485; IL11; ZNF121; BRD2; ZHX2; NCOA6; NOG; TFEB; FOS; BAZ1A; BAZ1B; PPRC1; SIRT1; PHF20L1; KAT2B; TOX2; SLTM; KIT; TET3; RARA; AHRR; ARHGEF2; ZNF236; JDP2; ZNF596; ZNF595; ZNF473; ZNF594; NR1I3; ZNF8; NFIL3; HSF4; MEF2D; ZNF585A; ZNF343; CDKN2B; ATAD2; GRHL1; VEGFA; PER2; PER1; NFIB; FOSB; ZNF699; ZNF578; ZNF577; ZNF697; GZF1; CCNT1; ZBTB21; ZBTB20; ARID4B; AHR; FGF2; ETS2; FOXQ1; ZNF84; POGZ; WWC3; ZNF568; KMT5A; ZNF324; ZNF202; FST; ZFP69B; ARID5A; ZBTB34; MITF; HIC1; ZNF91; DMTF1; ZNF93; ZNF317; ZNF316; ZNF557; ZNF674; ZNF551; KMT2D; ZNF550; DLX2; AKNA; KMT2A; KMT2C; GLIS3; FOXO3; ZNF429; SAFB2; ZKSCAN2; ZNF425; ZNF302; ZNF543; SKIL; ZNF300; ZNF783; BPTF; TFAP2A; AKIRIN2; ZFHX3; TFAP2E; SMAD9; INHBA; ESR2; SMAD7;*

	AHI1; NR4A1; CDK7; NR4A3; SP2; BCL6; SP4; MAFG; MAFF; RFXAP; JMY; PHF14; ZNF778; MDM4; MAFK; LHX4; MXD1; ZNF773; NFE2L1; NFE2L2; ZNF770; THRB; DDX3X; MIDEAS; AKAP8L; FHL2; HMGB2; CHD2; MYSM1; CHD1; PKD1; FLCN; ZNF408; ZNF528; ZNF768; ZNF644; KDM6B; KLF10; KLF11; MYOCD; TGIF2; ATRX; OLIG2; SAFB; ARID1A; KLF15; ETV5; ZNF75A; SARS1; CRY1; ZNF513; ZNF875; CREB5; NFAT5; CEBPB; UHRF1; CEBPG; ZBTB48; ZBTB43; NR2C2; HDAC9; ATAD2B; DLL4; ZNF749; ZNF507; E2F1; E2F2; E2F3; HES1; ZNF501; ZNF621; FNIP1; ZNF620; E2F7; E2F8; SPEN; TCF7L2; KDM4C; KLF4; TBX3; KLF6; KLF5; ZGLP1; KLF9; BCOR; ZNF852; LPIN1; SSBP2; TAF3; TNRC6A; TAF1
negative regulation of transcription by RNA polymerase II (GO:0000122)	*ZBTB21; BACH1; ETS2; MYC; SOX15; WWC3; EPC1; ZNF568; SOX8; KMT5A; ZNF202; MEF2A; FST; ZFP69B; ARID5A; MITF; DKK1; HIC1; RUNX1; DDIT3; ZNF557; TRIB3; ATF3; ZNF674; ATF4; ZNF550; ZNF274; DLX2; GATA6; ZNF20; GLIS3; NEDD4L; FOXO3; HIVEP1; ZNF425; ZBED6; SKIL; ZNF266; BPTF; TFAP2A; CREBBP; JUN; ZNF263; ZFHX3; GADD45A; LARP7; NR1D2; NR1D1; H1-0; ESR2; SOX30; SMAD7; SNW1; NR4A3; BCL6; SP2; SNAI1; SNAI2; ZNF778; GTF2IRD1; MAFK; MDM4; CPEB3; MXD1; EZH2; THRB; FHL2; FLCN; NIPBL; HEY1; ZMYM5; KLF10; KLF11; ZHX2; TGIF2; ATRX; NOG; SIRT1; ZNF75A; ETV5; SARS1; RARA; CRY1; ZNF875; JDP2; ZNF596; CEBPB; UHRF1; NR1I3; ZNF8; NR2C2; HDAC9; DLL4; NFIL3; ZNF749; E2F1; HSF4; HES1; FNIP1; ZNF620; E2F7; ZNF343; E2F8; SPEN; TCF7L2; KLF4; TBX3; VEGFA; PER2; PER1; NFIB; ZGLP1; NEDD4; FOSB; BCOR; TAF3; GZF1; TAF1*
positive regulation of transcription, DNA-templated (GO:0045893)	*ATF1; PID1; GABPB2; CCNT1; AHR; FGF2; ETS2; RPS6KA5; MYC; SOX15; ZXDC; POGZ; EPC1; SOX8; IER2; MEF2A; RFX3; MITF; RUNX2; RUNX1; LBHD1; DDX39B; DDIT3; ZSCAN21; HOXB3; PRKD1; ATF3; ATF4; KMT2D; ZNF395; AKNA; KMT2A; KMT2C; KMT2B; GATA6; GLIS3; XPC; FOXO3; PRDM10; NHLH2; ZNF827; HIVEP1; HIVEP3; PPARGC1A; ZNF300; ZBED3; BPTF; TFAP2A; EGR1; AKIRIN2; CREBBP; JUN; ZNF263; EGR2; XBP1; ZFHX3; JAG1; BRPF1; FZD4; SS18L1; EIF2AK3; LIF; NR1D2; INHBA; INO80; NR1D1; H1-0; ESR2; FOSL2; SOX30; SMAD7; FOSL1; AHI1; NR4A1; CDK7; SNW1; NR4A3; MAFF; RFXAP; REL; SNAI1; CREBRF; LHX4; NFE2L2; ZNF496; CSRNP1; DDX3X; THRB; AKAP8L; HMGB2; ZC3H8; AATF; BRCA2; MYSM1; PKD1; WDR43; ING5; FLCN; MED13; LBH; HEY1; MED10; CEP290; TEAD1; ARHGEF11; KDM6B; KLF10; IL11; MYOCD; NCOA6; ATRX; TFEB; AXIN1; FOS; PPRC1; ARID1A; SIRT1; KLF15; ETV5; KAT2B; DNAJC2; TOX2; IL1B; TET3; RARA; WAC; ZNF513; ARHGEF2; CREB5; NFAT5; CEBPB; UHRF1; NR1I3; ZBTB48; CEBPG; RNF6; NR2C2; ATAD2B; IFNL1; NFIL3; SERTAD1; SERTAD2; ZNF507; TP53INP2; HAS3; E2F1; E2F2; E2F3; HES1; MEF2D; E2F7; E2F8; MAP3K2; TCF7L2; CDKN2B; ATAD2; KLF4; GRHL1; TBX3; VEGFA; PER1; KLF6; RYBP; KLF5; NFIB; ZGLP1; NAA16; LPIN1; SSBP2*
negative regulation of transcription, DNA-templated (GO:0045892)	*ZBTB21; AHR; BACH1; ETS2; RPS6KA5; MYC; SOX15; WWC3; EPC1; ZNF568; SOX8; KMT5A; ZNF202; SAMD11; MEF2A; FST; SFMBT2; ZFP69B; ARID5A; RFX3; MITF; DKK1; HIC1; RUNX2; RUNX1; ZNF91; ZNF93; DDIT3; TRIB3; ZNF557; ATF3; ZNF674; ATF4; ZNF550; ZNF274; DLX2; L3MBTL3; GATA6; ZNF20; GLIS3; NEDD4L; FOXO3; MBTD1; HIVEP1; ZNF425; ZBED6; SKIL; ZNF266; BPTF; TFAP2A; CREBBP; JUN; ZNF263; ZFHX3; GADD45A; SETDB2; LARP7; NR1D2; NR1D1; H1-0; PHC3; ESR2; SOX30; SMAD7; SNW1; NR4A3; SP2; BCL6; SNAI1; SNAI2; MAP3K10; ZNF778; MDM4; GTF2IRD1; MAFK; MXD1; CPEB3; ZNF256; EZH2; THRB; MIDEAS; FHL2; HMGB2; ZC3H8; FLCN; NIPBL; SCML1; LBH; HEY1; ZMYM5; UIMC1; KLF10; KLF11; ZHX2; RBM15; TGIF2; NOG; SIRT1; ETV5; ZNF75A; SARS1; CRY1; RARA; ZNF875; JDP2; ZNF596; CEBPB; UHRF1; NR1I3; ZNF8; NR2C2; HDAC9; DLL4; BTAF1; IFNL1; NFIL3; ZNF749; E2F1; HSF4; HES1; FNIP1; ZNF620; E2F7; E2F8; ZNF343; SPEN; KDM4A; TCF7L2; KLF4; TBX3; VEGFA; PER2; PER1; ZGLP1; PDCD4; FOSB; BCOR; TAF3; GZF1; TAF1; CDK5R*
positive regulation of transcription by RNA polymerase II (GO:0045944)	*ATF1; PID1; GABPB2; CCNT1; AHR; FGF2; RPS6KA5; MYC; SOX15; POGZ; EPC1; SOX8; IER2; MEF2A; RFX3; MITF; RUNX2; RUNX1; DDIT3; ZSCAN21; HOXB3; PRKD1; ATF3; ATF4; KMT2D; ZNF395; AKNA; KMT2A; KMT2C; GATA6; GLIS3; FOXO3; PRDM10; NHLH2; TERT; ZNF827; HIVEP1; PPARGC1A; ZNF300; ZBED3; BPTF; TFAP2A; EGR1; AKIRIN2; CREBBP; JUN; ZNF263; EGR2; XBP1; ZFHX3; TGFB2; JAG1; SS18L1; LIF; LARP7; SMAD9; NR1D2; INHBA; INO80; NR1D1; FOSL2; SOX30; SMAD7;*

	FOSL1; AHI1; NR4A1; CDK7; SNW1; NR4A3; MAFF; RFXAP; REL; CREBRF; LHX4; NFE2L2; CSRNP1; DDX3X; THRB; AKAP8L; HMGB2; AATF; MYSM1; PKD1; FLCN; MED13; HEY1; MED10; TEAD1; KDM6B; KLF10; IL11; MYOCD; NCOA6; ATRX; TFEB; FOS; PPRC1; SIRT1; KLF15; ETV5; KAT2B; TOX2; TET3; RARA; ZNF513; ARHGEF2; NFAT5; CEBPB; UHRF1; NR1I3; CEBPG; NR2C2; ATAD2B; ZNF507; E2F1; E2F2; E2F3; HES1; MEF2D; E2F7; E2F8; TCF7L2; CDKN2B; ATAD2; KLF4; GRHL1; TBX3; VEGFA; PER1; KLF6; KLF5; NFIB; ZGLP1; LPIN1; SSBP2
regulation of gene expression (GO:0010468)	*MYLK2; PID1; JMJD1C; AHR; BACH1; FGF2; SLC8A1; CCAR1; FGF5; RIMS2; RPS6KA5; MYC; SOX15; POGZ; WWC3; ZNF562; FBXW7; SFMBT2; RC3H1; RFX3; MITF; ANK3; ATP1B1; PRKAB1; DKK1; HIC1; RUNX1; ZNF718; DMTF1; DDIT3; ZSCAN21; ZNF713; ZNF678; ADNP; ANKRA2; AJUBA; ATF4; ZNF430; KMT2D; ZNF274; ZNF395; YTHDC1; AKAP17A; PRDM10; PCBP3; TERT; ZNF827; STC2; PPARGC1A; SLC38A2; WNT3; ZNF146; FGF21; TFAP2A; TRAPPC2; EGR1; CREBBP; ZNF263; ZFHX3; TGFB2; TIPARP; DENND4A; BTN2A1; SETDB2; EIF2AK3; POU6F2; BRAF; NR1D2; INHBA; PUM1; CLK4; CLK3; ESR2; CLK1; OCLN; ZNF37A; BCL6; REL; GNB3; SNAI2; GTF2IRD1; EZH2; NFE2L2; LIN54; USP36; DDX3X; BTG1; CXCL8; ZNF492; CELF1; BRCA2; AFF4; FLCN; HEY1; TRIM4; ZNF280B; ZNF280C; NFKBIZ; ITGB8; AMH; ERMAP; KDM6A; KDM6B; TGIF2; ATRX; NOG; TFEB; NGF; F3; SIRT1; PTBP2; REXO4; SLC7A5; IL1A; IL1B; TRIM58; ZNF117; ZNF513; ZNF875; CEBPB; ZNF195; ROCK2; RNF6; NR2C2; PTHLH; DLL4; MAPK8; NFIL3; CAMK2N1; ZNF507; TDG; NOCT; ZNF506; E2F1; METTL4; ZNF502; KDM4A; WTIP; KLF4; VEGFA; PER1; RYBP; TRIM39; TRIML2; NOVA1; ADM2; ZNF736; SPRY2; SSBP2; TNRC6A; GZF1; TAF1*
intrinsic apoptotic signaling pathway in response to endoplasmic reticulum stress (GO:0070059)	*PPP1R15A; ERN1; BRSK2; CEBPB; DDIT3; BCL2; TNFRSF10B; ATP2A1; TRIB3; CHAC1; BBC3; ATF4*
intrinsic apoptotic signaling pathway (GO:0097193)	*PPP1R15A; MOAP1; BRSK2; CDKN1A; CEBPB; DDX3X; TNFRSF10B; ATP2A1; HIC1; BBC3; ERN1; DDIT3; JMY; TP53BP2; BCL2; E2F1; CYP1B1; PMAIP1; TRIB3; CHAC1; BCL2L2; ATF4; EPHA2*
regulation of cellular macromolecule biosynthetic process (GO:2000112)	*LIN54; USP37; BTG1; ZNF492; JMJD1C; AHR; BRCA2; BACH1; CCAR1; RPS6KA5; SOX15; ZNF280B; ZNF280C; WWC3; ZNF562; TGIF2; ATRX; TFEB; RFX3; MITF; HIC1; REXO4; ZNF718; DMTF1; DDIT3; ZSCAN21; ZNF713; ZNF117; MAPKAPK5; ZNF678; ZNF875; AJUBA; ATF4; ZNF430; KMT2D; ZNF274; ZNF395; CEBPB; ZNF195; RNF6; AKAP17A; FOXO3; NR2C2; ATXN2; NFIL3; ZNF506; E2F1; RBBP6; ZNF502; PPARGC1A; ZNF146; TRAPPC2; CREBBP; ZNF263; ZFHX3; DENND4A; POU6F2; NR1D2; WTIP; ESR2; RYBP; ZNF37A; ZNF736; SNAI2; GTF2IRD1; SSBP2; EZH2; GZF1*
**Enriched pathways with downregulated genes**	
cytokine-mediated signaling pathway (GO:0019221)	*CIITA; CD40; ITGAM; CSF1; TRADD; IFIT5; PIK3CD; ADAR; CXCL1; IFIT1; TNF; IFIT3; CX3CL1; CXCL5; IL27RA; MT2A; CCND1; TMSB4X; CFL1; MAP3K8; JAK3; TNFRSF4; TRIM21; EDARADD; EDN2; GSTO1; IL1R1; MMP3; TNFRSF1B; PRLR; MMP9; PSMA1; OAS2; OAS3; IRF2; FSCN1; LCP1; LTB; CARD14; IFNAR1; BIRC3; LAMA5; RNASEL; FOXC1; CEBPD; SHC1; EBI3; TNFRSF11B; IL2RG; SAMHD1; PSMB10; CNN2; MUC1; SOCS1; UBB; CCL2; GBP2; GBP1; MAPK3; STAT5A; TNFSF18; HSPA8; VCAM1; CCL20; STAT1; CD70; IL34; MX1; MSN; ISG15; PSMB8; PML; PSMB9; IL22RA1; NFKBIA; CXCL10; CXCL11; SAA1; TRIM31; MYD88*
chaperone cofactor-dependent protein refolding (GO:0051085)	*DNAJB1; HSPA8; SDF2L1; HSPA1L; DNAJB5; DNAJB4; HSPA6; HSPA2; HSPE1; HSPA1B; HSPA1A*
positive regulation of I-kappaB kinase/NF-kappaB signaling (GO:0043123)	*RNF31; CD40; CANT1; TRADD; IFIT5; LPAR1; SECTM1; CXXC5; TNF; LITAF; CX3CL1; LGALS1; CASP8; TSPAN6; TIFA; TNFSF10; TRIM21; IKBKE; LURAP1; APOL3; CD74; RHOC; FKBP1A; GPRC5B; TRIM14; ROR1; S100A4; CARD11; MYD88; TMEM106A; BIRC3*

'de novo' posttranslational protein folding (GO:0051084)	*DNAJB1; HSPA8; SDF2L1; HSPA1L; DNAJB5; DNAJB4; HSPA6; HSPA2; HSPE1; HSPA1B; HSPA1A*
negative regulation of viral process (GO:0048525)	*APOBEC3C; RNASEL; APOBEC3G; STAT1; MX1; IFIT5; ISG15; IFIT1; TNF; PML; PARP10; TNIP1; OAS2; OAS3; TRIM14; TRIM31; TRIM21*
regulation of I-kappaB kinase/NF-kappaB signaling (GO:0043122)	*RNF31; CD40; CANT1; TRADD; IFIT5; LPAR1; SECTM1; HSPB1; CXXC5; LITAF; TNF; CX3CL1; LGALS1; CASP8; TSPAN6; TIFA; TNFSF10; TRIM21; IKBKE; LURAP1; APOL3; CD74; STAT1; RHOC; FKBP1A; GPRC5B; TNIP1; TNIP3; TRIM14; ROR1; S100A4; TMEM106A; CARD11; MYD88; BIRC3*
cellular response to type I interferon (GO:0071357)	*RNASEL; STAT1; MX1; IFIT5; ADAR; ISG15; IFIT1; SAMHD1; IFIT3; PSMB8; OAS2; OAS3; IRF2; GBP2; MYD88; IFNAR1*
type I interferon signaling pathway (GO:0060337)	*RNASEL; STAT1; MX1; IFIT5; ADAR; ISG15; IFIT1; SAMHD1; IFIT3; PSMB8; OAS2; OAS3; IRF2; GBP2; MYD88; IFNAR1*
mitochondrial respiratory chain complex I assembly (GO:0032981)	*DUFB7; DMAC1; NDUFB6; NDUFB5; NDUFB4; NDUFA3; NDUFC2; NDUFB1; NDUFC1; NDUFAF8; TMEM126A; NDUFAF3; TMEM126B; NDUFV3; NDUFAF1*
NADH dehydrogenase complex assembly (GO:0010257)	*NDUFB7; DMAC1; NDUFB6; NDUFB5; NDUFB4; NDUFA3; NDUFC2; NDUFB1; NDUFC1; NDUFAF8; TMEM126A; NDUFAF3; TMEM126B; NDUFV3; NDUFAF1*
